# Effects of icariin on the proliferation and osteogenic differentiation of human amniotic mesenchymal stem cells

**DOI:** 10.1186/s13018-020-02076-9

**Published:** 2020-12-02

**Authors:** Fang Wang, Zhiyong Yang, Wei He, Qinggao Song, Kun Wang, Yali Zhou

**Affiliations:** 1 Oral Maxillofacia Trauma and Orthognathic Surgery, Hospital of Stomatology, Zunyi Medical University, Zunyi, Guizhou China; 2 Oral Maxillofacia Trauma and Orthognathic Surgery, the First Hospital of Changsha, Changsha, Hunan China

**Keywords:** Human amniotic mesenchymal stem cell, Icariin, Proliferation, Osteogenic differentiation

## Abstract

**Background:**

Tissue engineering technology has been applied extensively for clinical research and human amnion mesenchymal stem cells (hAMSCs) could cause mesenchymal stem cells to differentiate into the bone tissue. However, it is necessary to develop and identify the safer appropriate amount of osteogenic inducer. The objective of this study is to investigate the effect of icariin (ICA) on the proliferation and osteogenic differentiation of hAMSCs.

**Methods:**

The morphology and phenotype of hAMSCs were discovered by flow cytometry and immunocytochemical staining. The osteogenic differentiation of hAMSCs under the influence of different concentrations of ICA were assessed by alkaline phosphatase (ALP) activity substrate assay and alizarin red staining.

**Results:**

MTT assay revealed that the hAMSCs pretreated with ICA exhibited increased proliferation when compared with the control group, and the most optimum concentration of ICA was 1 × 10^− 6^ mol/L. The combined analysis of ALP activity and ARS staining showed that ICA could significantly promote the osteogenic differentiation of hAMSCs, and the effect was most significant when the concentration of ICA was 1 × 10^− 6^ mol/L.

**Conclusion:**

All the above results implied that ICA could significantly increase proliferation and enhance the osteogenic differentiation of hAMSCs, especially when the concentration of ICA was 1 × 10^− 6^ mol/L.

## Background

Bone defects/losses is a serious problem in orthopedics due to low healing rate using traditional treatment and increases patients’ pain and heavily burdens families [1, 2]. Over the last two decades, tissue engineering technology has been applied extensively for clinical research. For example, stem cell-based tissue regeneration showed certain curative effects as a novel curative therapy for bone diseases. However, methods for collection, isolation, and maintenance of stem cells in vitro is still a challenge of stem cell-based clinical healing for bone disorder, which is also disadvantageous to reduce operation time [3]. Moreover, it is still a worry whether stem cell would differentiate into bone-forming osteoblasts. Although the osteogenic inducers such as bone morphogenetic proteins (BMP) and vascular endothelial growth factor (VEGF) are important factors for healing of bone defects/losses, there are still some disadvantages for low differentiation efficiency, complicated formulas, and high cost [4]. Therefore, the key point is to identify safe and effective osteogenic inducers to improve stem cell-based therapy for bone regeneration.

Previous findings showed that some drugs including statins [5], isoflavone derivatives [6, 7], and TAK-778 [8] could regulate the balance in bone formation between osteoblastic bone formation and osteoclastic bone resorption [9]. Recently, many traditional Chinese medicines were used for fractured bone healing, and some natural small molecule compounds isolated from them were confirmed as effective osteogenic inducers. Icariin (ICA), as the main active compound of *Epimedium pubescens*, was reportedly used to cure bone diseases in ancient China [10]. And it could promote an agent in cartilage tissue engineering [11] and repair of articular cartilage defects in rabbit knees [12]. It has been found the beneficial effects of ICA is to exert an inducing function on the osteogenic differentiation [13] and stimulate the bone marrow-derived mesenchymal stem cell (BMSC) proliferation in stem cell therapy [14]. Therefore, ICA is expected to be a safe and effective osteoinductive active factor for clinical bone regeneration.

Human amniotic mesenchymal stem cells (hAMSCs), derived from ditched amniotic membrane (AM) [15, 16], are a center of attention in mesenchymal stem cells (MSCs) for bone stem cell-based tissue engineering and regenerative medicine (TE&RM) use owing to their superior properties [17]. HAMSCs have unique advantages for bone regeneration in a noninvasive way and have immunomodulatory properties with weak anti-inflammatory characteristics, high differentiation, and without ethical controversy [18]. In addition, previous studies found that hAMSCs have been successfully differentiated into osteoblasts using the classic tri-component osteogenic medium and promote bone regeneration in vivo and in vitro [19]. In this study, we aim to make clear effects of icariin on the proliferation and osteogenic differentiation of human amniotic mesenchymal stem cells.

## Materials and methods

### Cell isolation, cultivation, and identification of hAMSCs

The placental amniotic tissue was collected from normal pregnant women undergoing full-term cesarean section after gaining the informed consent of the pregnant women or her relatives. This project was approved by the medical ethics committee of Zunyi Medical University (Approval No. 2018. 246). After removing residual blood under sterile conditions, the amniotic tissue was cut into small pieces of 1–2 cm^2^ and then separated and repeatedly rinsed using phosphate-buffered saline (PBS) containing 100 μg/mL streptomycin and 100 U/mL penicillin (Beyotime, China). Amnion fragments were collected in a 50 mL centrifuge tube and digested for 35 min at 37 °C in a double volume of digestion solution I (0.05% trypsin containing 0.02% EDTA-2Na). After centrifugation at 180 rpm for 2 h at 37 °C, the remaining amnion in the centrifuge tube was filtered through a filter screen and digested by a double volume of digestion solution II (0.75 mg/mL collagenase II containing 0.075 mg/mL DNase I) to acquire hAMSCs. The sample was shaken at a speed of 180 r/min and digested for approximately 2 h at 37 °C. After filter and centrifuge, the resultant hAMSCs were transferred to low-glucose (LG)-DMEM complete medium, supplemented with 10% heat-inactivated fetal bovine serum (FBS) to rest the cell suspension in a humid atmosphere of 5% CO_2_ for 5 min at 37 °C to precipitate hAMSC (passage 0, P0).

The resultant hAMSCs (passage 0, P0) were collected in a 50-mL centrifuge tube and digested for 3 min at 37 °C in a double volume of digestion solution I (0.05% trypsin containing 0.02% EDTA-2Na). When the cells reached 80% confluence, they were digested and subcultured into flasks. Cells from passage 3 (P3) were used for further analysis in this study. The cell morphology was constantly observed under a light microscope (Olympus, Tokyo, Japan). In addition, these hAMSCs were analyzed using the immunocytochemical staining method according to the protocols as described in a previous study [20].

For the phenotypic characterization of hAMSCs, P3 hAMSCs were trypsinized and subsequently washed with D-PBS containing 0.1% BSA, adjusted to a density of 1.5 × 10^6^ cells/mL, and then incubated with and HLA-DR for 25 min in the dark. After washing again with D-PBS containing 0.1% BSA, the cell suspension was centrifuged at 1000 rpm for 5 min and the supernatant was discarded. Finally, the labeled cells were analyzed using the flow cytometry system (FACS Calibur, Becton Dickinson, USA) and CellQuest software (BD, NJ, USA) after fixation with 1% paraformaldehyde.

### Cell proliferation assay

This experiment was divided into three groups: blank group (no cells and no ICA), control group (with cells but no ICA), and ICA group (with cells and ICA). The P3 hAMSCs were seeded at a density of 1 × 10^4^ cells/mL in 96-well plates. After incubation for 24 h, the medium was changed to ICA-containing media at a concentration of 0 (blank and control), 1 × 10^− 4^ (ICA-1), 1 × 10^− 5^ (ICA-2), 1 × 10^− 6^ (ICA-3), 1 × 10^− 7^ (ICA-4), and 1 × 10^− 8^ (ICA-5) mol/L accordingly. Cells were incubated at 37 °C in a 5% CO_2_ incubator for 72 h, and 20 μL 5 mg/mL MTT was then added to each well, followed by incubation at 37 °C in a 5% CO_2_ incubator for 4 h. Later, the medium was discarded, and 150 μL of dimethylsulfoxide (DMSO) was added to each well. After incubation at 37 °C for 15 min, the OD value of each well was determined at a wavelength of 490 nm by a microplate reader (MultiskanTM GO, ThermoFisher, Waltham, MA, USA).

### Osteogenic differentiation of hAMSCs in vitro

The P3 hAMSCs were seeded at a density of 1 × 10^5^ cells/mL in 96-well plates in LG-DMEM culture medium under sterile conditions. After 24 h of incubation, the medium was replaced with LG-DMEM culture medium with 10% FBS. The experiment was divided into 5 groups according to the different substances added to the medium, namely, the classic group (100 nmol/L dexamethasone (Sigma, SL, USA), 50 mg/L vitamin C (Solarbio, Beijing, China), and 10 mmol/L β-glycerophosphate (Solarbio, Beijing, China)), the blank group (without additives), the ICA-1 group (1 × 10^− 4^ mol/L ICA), the ICA-2 group (1 × 10^− 5^ mol/L ICA), and the ICA-3 group (1 × 10^− 6^ mol/L ICA). Cells were incubated at 37 °C in a 5% CO_2_ incubator for 72 h, and 20 μL 5 mg/mL MTT was then added to each well, followed by incubation at 37 °C in a 5% CO_2_ incubator for 4 h. The culture medium was changed every 3 days, and the intervention was continuously induced for 21 days. Meanwhile, during the induction period, the cell morphology of the five periods of 1, 3, 7, 14, and 21 days were photographed for morphological observation, and the samples were preserved. ALP (alkaline phosphatase) was extracted and detected with the ALP assay kit (AnaSpec, San Jose, CA) as directed by the manufacturer. The cells on the 21st day were stained with alizarin red staining (ARS), and statistical analysis was performed based on the number of stained calcium nodules.

### Statistical analysis

Experimental data were expressed as mean ± standard deviation (SD) and analyzed using the SPSS 19.0 statistical software. One-way analysis of variance was used for comparison between groups, and the rate comparison was performed by *χ*^2^ test. *P* < 0.05 was considered as statistically significant.

## Results

### Morphology and phenotype of hAMSCs

**Fig. 1:**
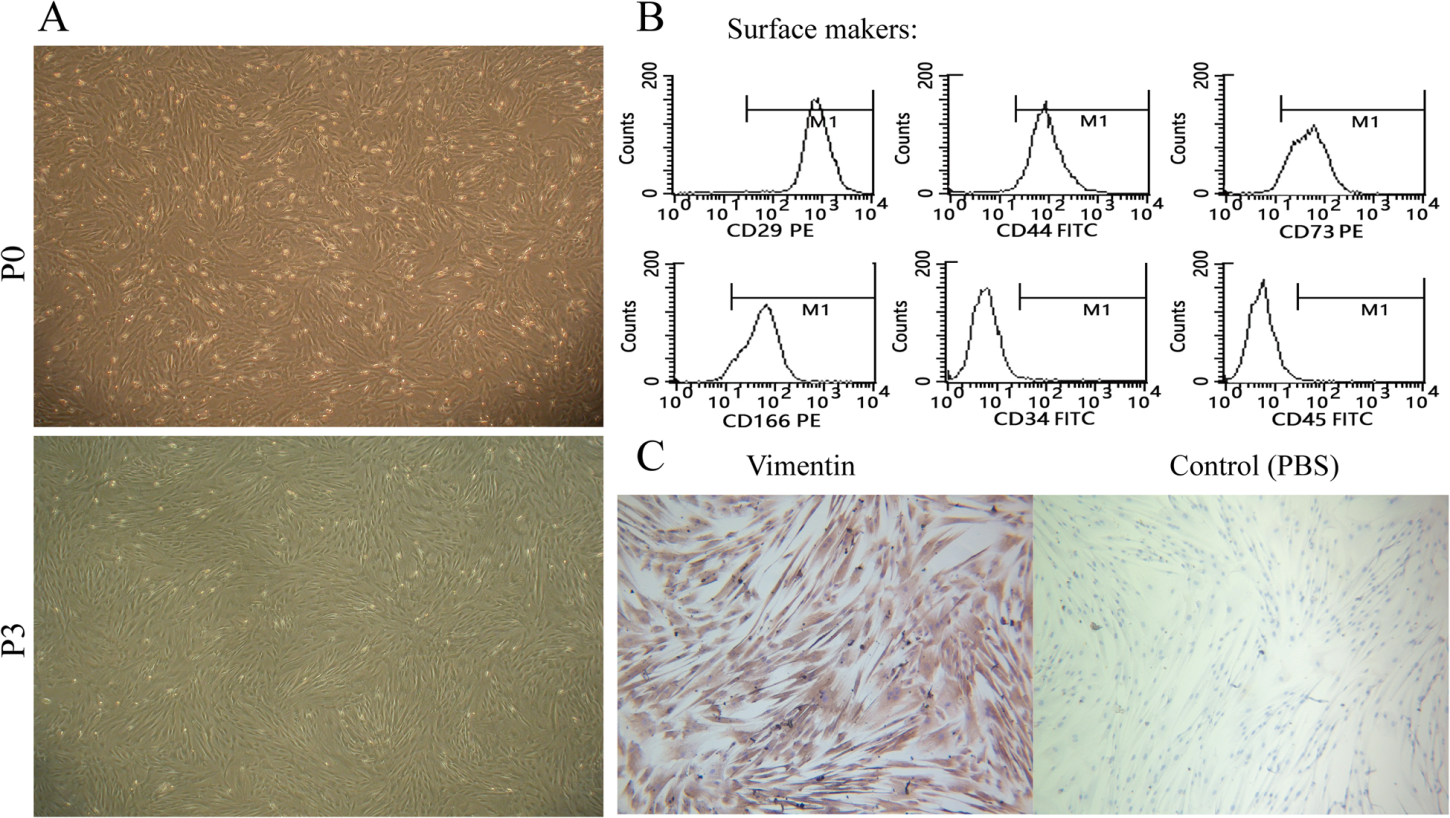
Morphology and phenotype of hAMSCs. **a** Morphology of P0 and P3 hAMSCs. **b** Expression of hAMSC surface markers. **c** Expression of skelemin proteins of hAMSCs

The microscopic examination of P0 hAMSCs cultured in vitro revealed a typical adherent growth state (Fig. 1a). After 48 h of primary culture, the majority of the adherent cells stretched and became spindle- or short-rod-shaped. With the increase of passage number, small quantities of epithelial cells and dead cells disappeared rapidly. When the hAMSCs were sub-cultured to the third passage, they displayed a fibroblast-like morphology in radial or whirlpool patterns (Fig. 1a).

Flow cytometry revealed that hAMSCs highly expressed MSC surface markers CD29, CD44, CD73, and CD166 (Fig. 1b). On the other hand, very low expressions of cell surface markers belonging to CD34 and CD45 were showed (Fig. 1b). ICC staining indicated that hAMSCs also highly expressed mesenchymal cell marker vimentin, which proved that hAMSCs were of human origin and a member of the MSC family (Fig. 1c).

### Effect of different concentrations of ICA on the proliferation of hAMSCs

**Fig. 2:**
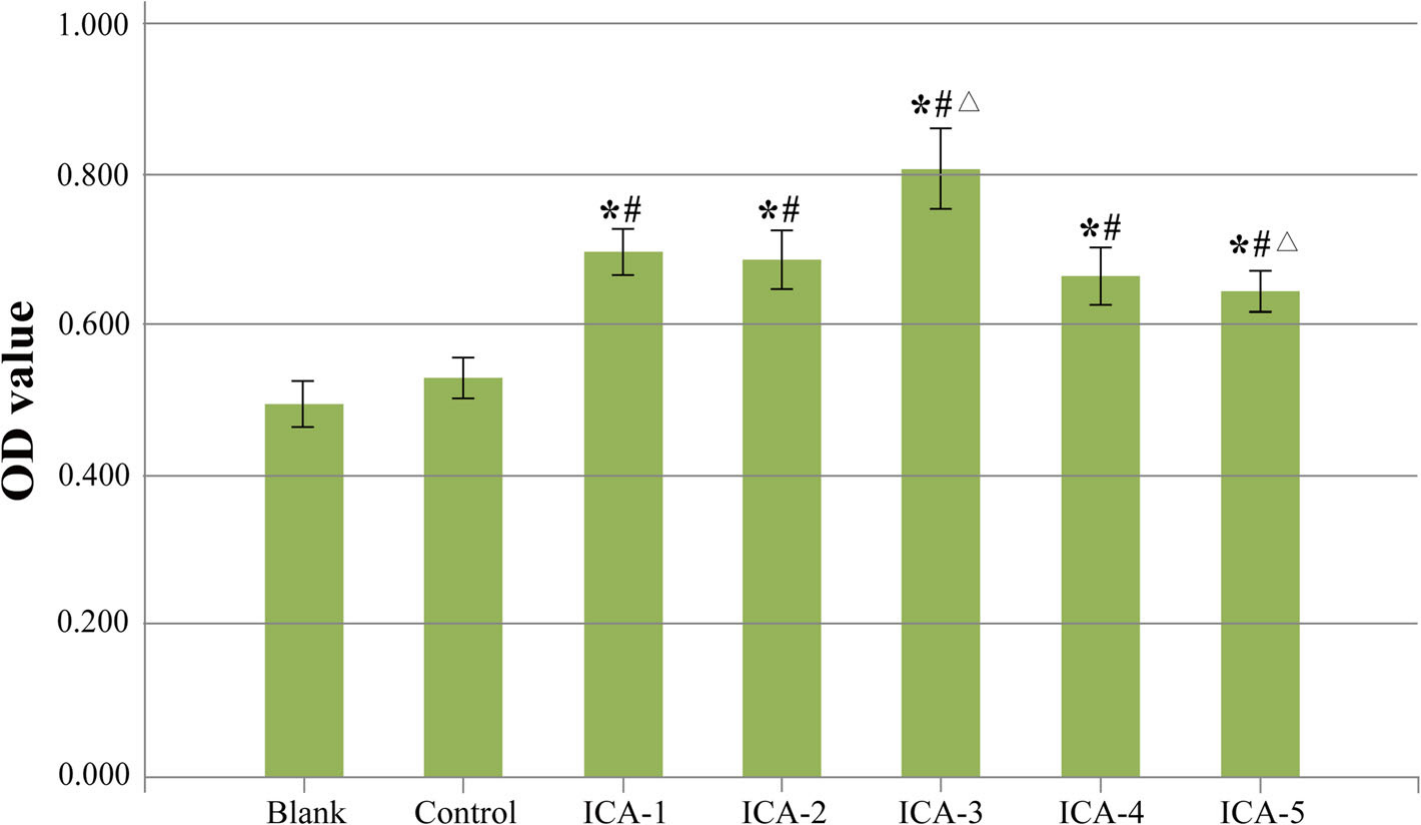
Exogenous ICA promotes the proliferation of hAMSCs in vitro. Compared with the control group, ^*^P < 0.05; compared with the blank group, ^#^*P* < 0.05; and compared with the ICA-1, ICA-2, ICA-4, and ICA-5 groups, ^△^*P* < 0.05

The stimulation effect of ICA on the proliferation of hAMSCs during 3 days of culture was evaluated at various concentrations (1 × 10^− 4^ mol/L, 1 × 10^− 5^ mol/L, 1 × 10^− 6^ mol/L, 1 × 10^− 7^ mol/L, 1 × 10^− 8^ mol/L). OD absorbance values of samples cultured for different times are measured to evaluate the proliferation kinetics of osteoblast-like cells. MTT assay revealed that the hAMSCs pretreated with ICA exhibited increased proliferation when compared with the control group. Meanwhile, in the presence of ICA, the proliferation fold of hAMSCs increases first and then decreases with the increase of ICA concentration. For example, ICA increased the proliferation from 0.524 ± 0.033-fold in the control group to 0.728 ± 0.048 (*P* < 0.05), 0.719 ± 0.044 (*P* < 0.05), 0.688 ± 0.057 (*P* < 0.05), and 0.659 ± 0.041-fold (*P* < 0.05) at 1 × 10^− 4^, 1 × 10^− 5^, 1 × 10^− 7^, and 1 × 10^− 8^ mol/L, respectively. It is noteworthy that ICA at the optimum concentration of 1 × 10^− 6^ mol/L could increase the proliferation of hAMSCs from 0.524 ± 0.033-fold to 0.807 ± 0.080-fold (*P* < 0.05) compared with the control group (Fig. 2).

### Effect of different concentrations of ICA on the osteogenic differentiation in hAMSCs

**Fig. 3:**
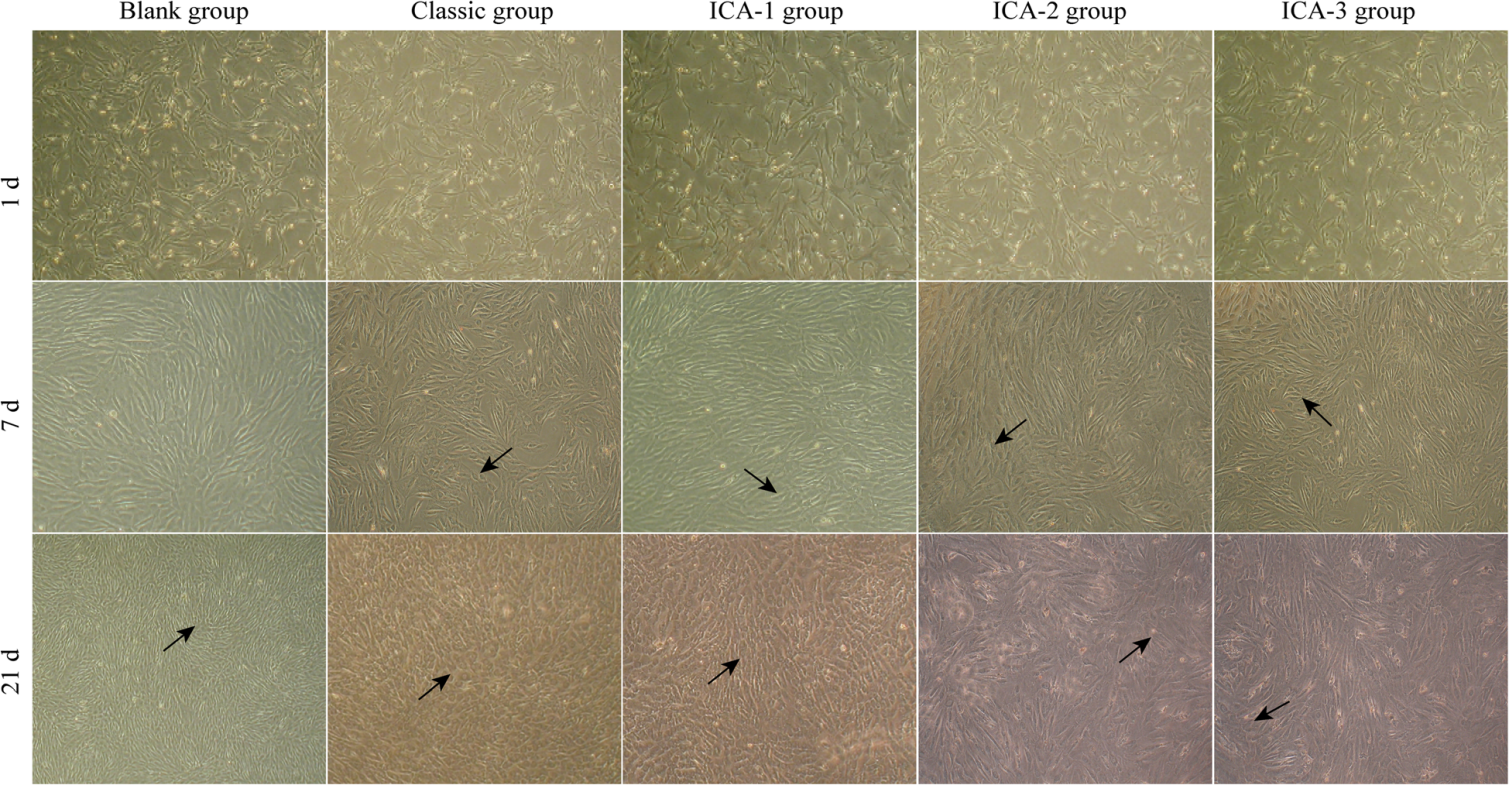
Morphological changes of hAMSCs under the effect of ICA on the 7th, 14th, and 21st day

To determine the effect of different concentrations of ICA during hAMSC osteogenic differentiation, the cell morphology at different stages of 1, 7, and 21 days were photographed for morphological observation. As shown in Fig. 3, the cells in each group basically showed a long spindle shape on day 1. Following a 7-day induction period, the morphology of hAMSCs in classic and ICA groups changed from long spindle to polygonal and triangular (Fig. 3). Twenty-one days post-osteoinduction, hAMSCs in both the classic group and the ICA group indicated morphological changes: the cells transitioned from the typical long-spindle morphology to short-spindle, triangle, or polygonal shapes with clear boundaries between the cells (Fig. 3).

**Fig. 4:**
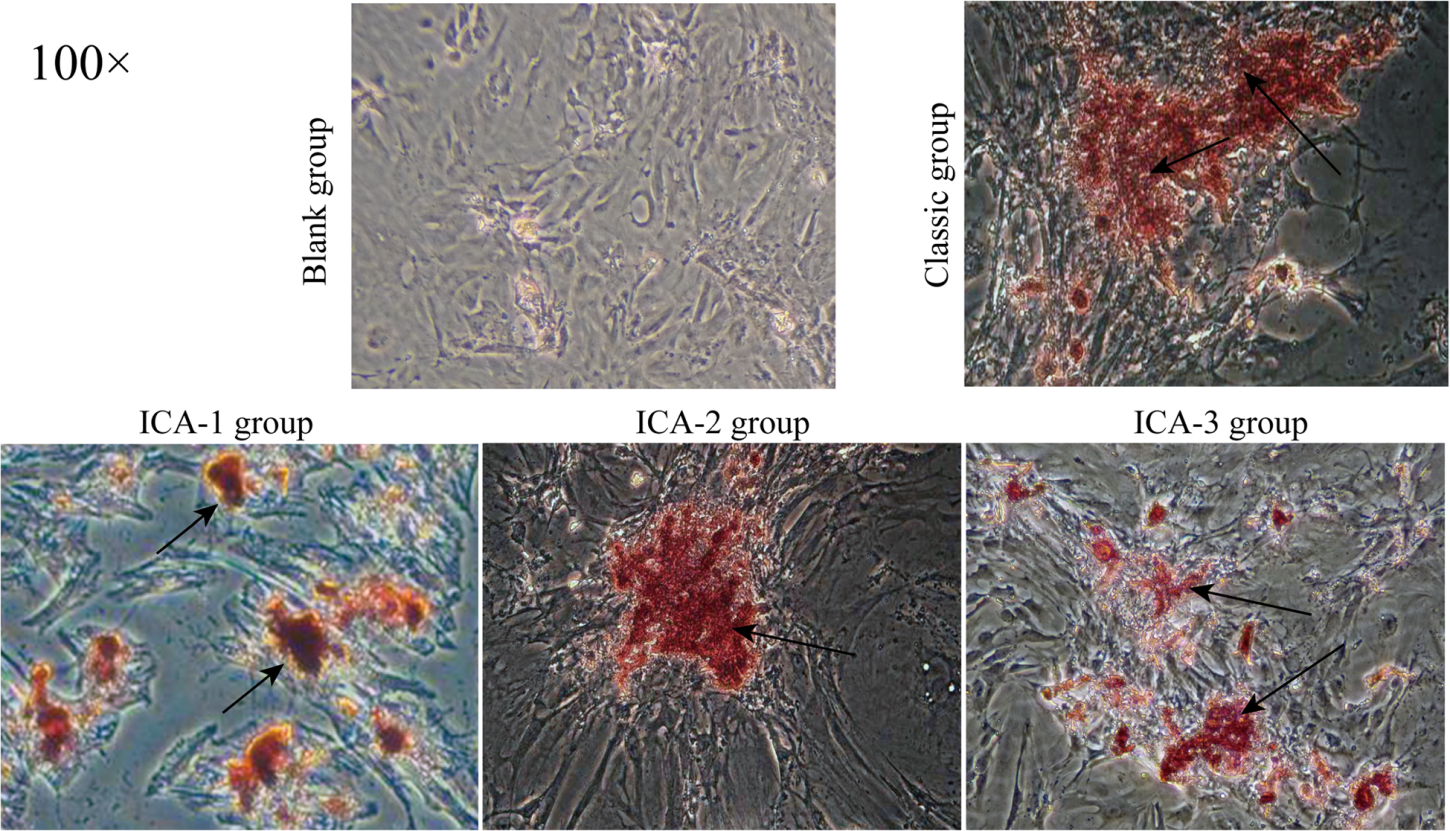
ALP expression as assessed by ARS staining on day 21

The marker of bone differentiation is that the osteogenic differentiation of MSC leads to mineralization or the formation of osteoblast nodules. Thus, on day 21 of the osteogenic induction experiment, the formation of mineralized nodules in hAMSCs was evaluated by alizarin red staining (ARS) and the amount of mineralization was quantified by eluting ARS staining from differentiated osteoblasts. As shown in Fig. 4, the treatment in blank group had no nodules formed to minimal ARS staining, indicating that the cells in the blank group did not show mineralization. The cells in the classic group exhibited characteristic osteoblast nodules (Fig. 4). In addition, numerous rose-red calcified nodules in ARS staining were observed in the ICA-1, ICA-2, and ICA-3 groups compared with the blank group, and the most ARS staining was found in the ICA-3 group (Fig. 4). This was confirmed by quantitative analysis of ARS, as shown in Table S1.

### Effect of different concentrations of ICA on ALP activity of hAMSCs

**Fig. 5:**
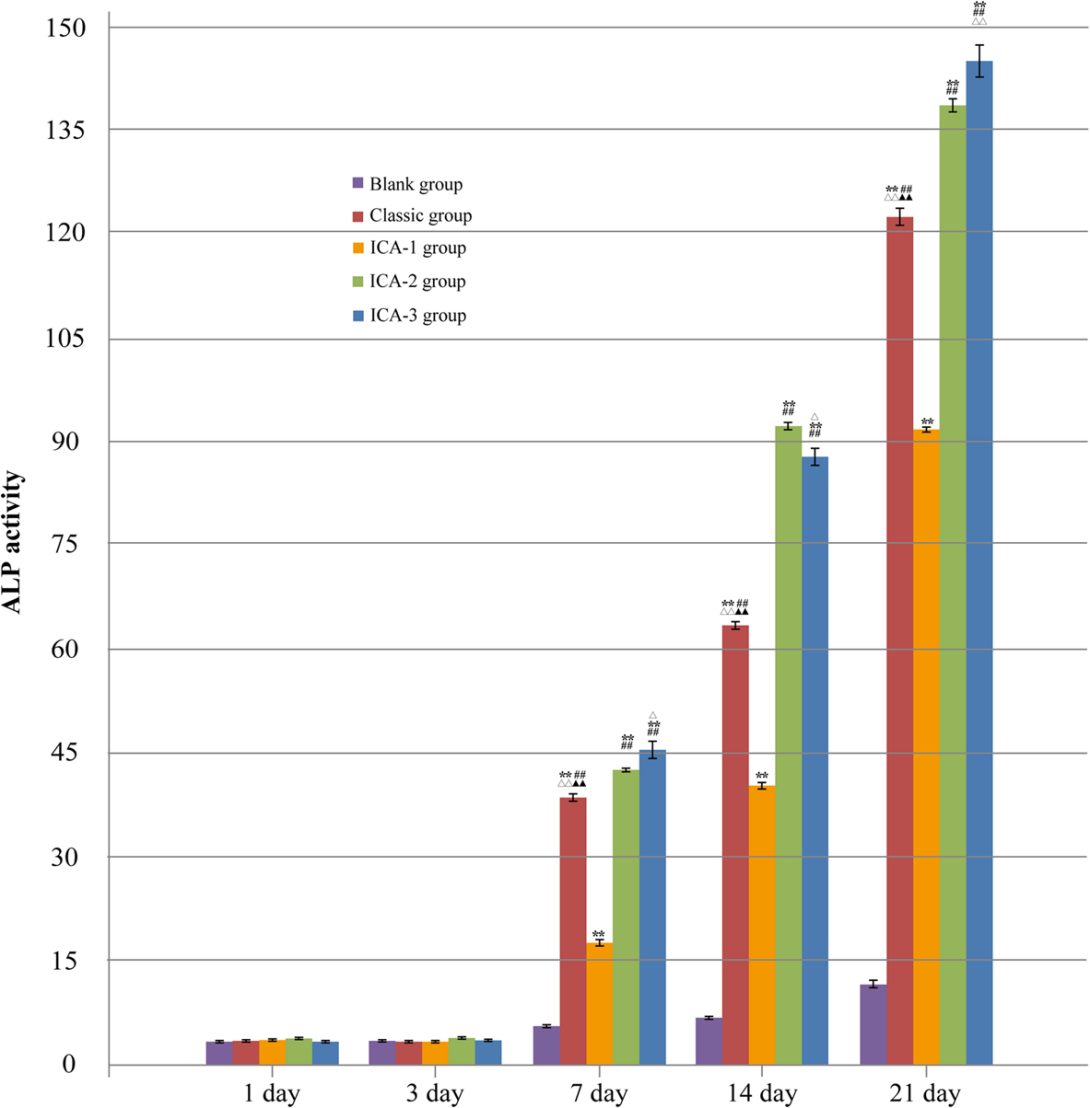
ALP activity of hAMSCs after ICA treatment on days 1, 3, 7, 14, and 21. Compared with the blank group, ^**^P < 0.01; compared with the ICA-1 group, ^##^*P* < 0.01; compared with the ICA-2 group, ^△△^*P* < 0.01, ^△^*P* < 0.05; and compared with the ICA-3 group, ^▲▲^*P* < 0.01

ALP activity usually reflects the early osteogenic differentiation of hAMSCs. The investigation of ALP activity was done after hAMSCs had been cultured for 1, 3, 7, 14, and 21 days. The ALP activity was not significantly different compared with the blank group, and the cells did not show obvious differentiation on days 1 and 3 of induction (Fig. 5). From 7 to 21 days of cultivation, the activity of ALP rose and presented a significantly higher level in the classic group and ICA group than in the blank group. After 7 days of cultivation, compared to the blank group (5.614 ± 0.377), the ALP activity of hAMSCs in the ICA-1, ICA-2, ICA-3, and classic group reached 17.491 ± 0.509 (*P* < 0.01), 43.073 ± 0.422 (*P* < 0.01), 45.534 ± 1.072 (*P* < 0.0he 5), and 38.598 ± 0.6444 (*P* < 0.01). ALP activity in the ICA-1, ICA-2, ICA-3 group (41.656 ± 0.375, 92.222 ± 0.661, 88.195 ± 1.908), and the classic group (63.583 ± 0.615) were significantly higher than that in the blank group (7.457 ± 0.377) on the 14th day. Following a 21-day culture period, the ALP activity of hAMSCs in ICA-1, ICA-2, ICA-3, and the classic group reached 92.819 ± 0.509 (*P* < 0.01), 139.955 ± 1.428 (*P* < 0.01), 145.102 ± 2.384 (*P* < 0.01) and 122.056 ± 1.676 (*P* < 0.01) compared to the blank group (11.175 ± 0.799) (Fig. 5).

## Discussion

Osteogenic inducers are very important for effective stem cell-based treatment of bone defects/losses. Recently, many researches showed that traditional Chinese medicines could induce directional MSC differentiation into osteoblasts [21–25]. These traditional Chinese medicines such as *Salvia miltiorrhiza Bunge*, *Angelica sinensis*, *Astragalus membranaceus Bunge*, *Puerarin radix*, and *Epimedium* spp. may be used to promote osteogenesis and inhibit bone resorption, showing that they had a positive effect on the treatment of bone defects/losses [9, 26]. Icariin (ICA) is the natural main active product of *Epimedium pubescens* which is widely used in traditional Chinese medicine (TCM) and could promote bone formation by stimulating osteogenic differentiation of MSC recently [9, 10, 27–33]. ICA could activate the Wnt/β-catenin signaling pathway to promote chondrogenic differentiation [27]. In addition, activating ERK and p38 MAPK signaling achieved rat BMSC proliferation and increased the phosphorylation level of GSK-3β and cyclinD1 protein [28, 29]. Some researches showed that icariside II (ICA II) is a kind of metabolite of ICA (loss of the glycosyl moiety at the C-7 position of ICA) which increased ALP activity and calcium deposition to enhance the osteogenic differentiation of BMSCs via enhanced expression of osteogenesis proteins/genes (Runx2, collagen I), increased the PI3K/AKT/mTOR/S6K1 signaling pathways, and downregulated PPARγ, Fabp4, and adipsin gene expression [30–33]. Based on the above researches, we further explore optimum concentrations of ICA on proliferation and osteogenic differentiation of hAMSCs and our results verified that the potent osteogenic effect on hAMSCs was induced by icariin. Therefore, ICA will be a hotspot for bone regenerative medicine due to the extremely low-cost compound and its high abundance.

Although ICA occupied multiple advantages on the proliferation and osteogenic differentiation for bone disease, they might be unexpectedly cytotoxic against stem cells due to a dose-response relationship. In this study, the stimulation effect of ICA on the proliferation of hAMSCs during 3 days of culture was evaluated at various concentrations with 1 × 10^− 4^mol/L, 1 × 10^− 5^ mol/L, 1 × 10^− 6^ mol/L, 1 × 10^− 7^ mol/L, and 1 × 10^− 8^ mol/L. MTT assay indicated that the hAMSCs pretreated with ICA exhibited increased proliferation compared with the control group, and the proliferation fold of hAMSCs decreases with the increase of ICA concentration (Fig. 4). Previous studies showed that 0.1–10 μM icariin stimulated the proliferation of rat bone marrow stromal cells (rMSCs) and increased the alkaline phosphatase activity, osteoalcin secretion, and calcium deposition level of rMSCs during osteogenic induction [34]. Wang et al. (2016) investigated the concentration of 5 × 10^− 6^ mol/L for ICA on chondrogenic differentiation of bone marrow stromal cells by Wnt/β-catenin signaling pathway [27]. Song et al. (2013) estimated the effect of ICA on osteoblast proliferation and found that compared with the control group, various doses of icariin (0.1–100 nM) could significantly increase the cell number and the 10 nM concentration dramatically increased osteoblast differentiation and mineralization [35]. In this study, ICA at the optimum concentration of 1 × 10^− 6^ mol/L could increase the proliferation of hAMSCs via MTT array. In addition, ARS staining, the formation of mineralized nodules, and ALP activity altogether showed a concentration of 1 × 10^− 6^ mol/L of ICA could induce osteoblast differentiation. Therefore, 1 × 10^− 6^ mol/L ICA showed the highest cell viability and osteogenic activity on hAMSCs in the present study, indicating that osteogenic hAMSC induction is sensitive to ICA.

Alkaline phosphatase (ALP) is one of the biochemical markers for osteoblast activity. It is an enzyme in which osteoblasts secrete into the extracellular matrix [36]. ALP can catalyze the hydrolysis of the phosphate esters in an alkaline environment, resulting in the formation of an inorganic phosphate that plays an important role in bone mineralization [37]. Besides, at the processes of the extracellular matrix through the osteoblast, calcium is one of the components and the investigation of calcium deposition becomes remarkable as an indicator of in vitro activities of the osteoblast [38]. Alizarin red staining (ARS), as a marker of calcium deposition in these processes, has been used in many related studies to assess mineralized matrix deposition for osteoblast [9, 38, 39]. In this study, the activity of ALP rose and presented a significantly higher level of activity in the classic group and ICA groups with different concentrations of ICA than in the blank group from 7 to 21 days of hAMSC cultivation (Fig. 5). At the same time, numerous rose-red calcified nodules in ARS staining were observed in the classic group and ICA groups (Fig. 4). Our study confirmed that ICA would promote hAMSC differentiation into osteoblast by ALP activity and ARS staining together.

## Conclusions

Bone defects/losses is a serious problem in orthopedics, and osteogenic inducers play central roles in effective stem cell-based treatment during the processes. In this study, we have demonstrated that icariin is a safe, effective, and novel natural osteogenic inducer for hAMSCs. When the concentration of ICA was 1 × 10^− 6^ mol/L, it is most significant to increase proliferation and promote the osteogenic differentiation of hAMSCs. Therefore, an appropriate amount of icariin might be used as a potential candidate compound for stem cell-based therapy of bone disease.

## Supplementary Information

**Additional file 1: Table S1.** Number of calcified nodules on days 21 in each group.

## Data Availability

The datasets used and/or analyzed during the current study are available from the corresponding author on reasonable request.

## Abbreviations

ALP: Alkaline phosphatase
ARS: Alizarin red staining
DMEM: Dulbecco’s modified Eagle’s medium
DMSO: Dimethyl sulfoxide
FBS: Fetal bovine serum
hAMSCs: Human amnion-derived mesenchymal stem cells
ICA: Icariin
MTT: 3-(4,5-Dimethylthiazol-2-yl)-2,5-diphenyltetrazolium bromide
BMP: Bone morphogenetic proteins
VEGF: Vascular endothelial growth factor
BMSCs: Bone marrow-derived mesenchymal stem cells
MSCs: Mesenchymal stem cells
TE&RM: Tissue engineering and regenerative medicine
AM: Amniotic membrane
PBS: Phosphate-buffered saline
rMSCs: Rat bone marrow stromal cells
