# Supplementary material for: Effects of icariin on the proliferation and osteogenic differentiation of human amniotic mesenchymal stem cells
Source: J Orthop Surg Res. 2020 Dec 2;15:578. doi: 10.1186/s13018-020-02076-9 (PMC7709318; doi:10.1186/s13018-020-02076-9)
Supplement: Supplementary file 1 — Additional file 1: Table S1. Number of calcified nodules on days 21 in each group. [file 13018_2020_2076_MOESM1_ESM.docx]

Table S1. Number of calcified nodules on days 21 in each group

| Group | calcified nodules |
| --- | --- |
| Blank | 0.667 ± 0.577 |
| Classic | 4.333 ± 1.527^##^ |
| ICA-1 (1×10^-4^ mol/L) | 1.333 ± 0.577 |
| ICA-2 (1×10^-5^ mol/L) | 4.666 ± 2.08^##^ |
| ICA-3 (1×10^-6^ mol/L) | 5.333 ± 0.577^##**^ |

Compared with blank group, ^##^*P* < 0.01; compared with ICA-1 and ICA-2 group, ^**^*P* < 0.01
